# Improvement of Sludge Dewaterability by Ultrasound-Initiated Cationic Polyacrylamide with Microblock Structure: The Role of Surface-Active Monomers

**DOI:** 10.3390/ma10030282

**Published:** 2017-03-13

**Authors:** Chuanliang Zhao, Huaili Zheng, Li Feng, Yili Wang, Yongzhi Liu, Bingzhi Liu, Badradine Zakaria Djibrine

**Affiliations:** 1 Key Laboratory of the Three Gorges Reservoir Region’s Eco-Environment, State Ministry of Education, Chongqing University, Chongqing 400045, China; zhaochuanliang90@sina.com (C.Z.); fl19860314@126.com (L.F.); zhaocl90@126.com (Y.L.); 15223019928@163.com (B.L.); badradinedjibrine@foxmail.com (B.Z.D.); 2 National Centre for International Research of Low-carbon and Green Buildings, Chongqing University, Chongqing 400045, China; 3 College of Environmental Science and Engineering, Research Center for Water Pollution Source Control and Eco-remediation, Beijing Forestry University, Beijing 100083, China; wangyilimail@126.com

**Keywords:** cationic polyacrylamide, surface-active monomer, sequence distribution, microblock structure, sludge dewatering, floc properties, soluble extracellular polymeric substances

## Abstract

Cationic polyacrylamides have been employed widely to improve sludge dewatering performance, but the cationic units are randomly distributed in the molecular chain, which restricts the further enhancement of dewaterability. Common template technology to prepare block copolymers requiring a huge number of templates reduces the polymer purity and molecular weight. Here, we adopted the surface-active monomer benzyl dimethyl 2-(methacryloyloxy)ethyl ammonium chloride (BDMDAC) to synthesize cationic microblocky polyacrylamide initiated by ultrasound. The reactivity ratio of monomers suggested that novel cationic monomer BDMDAC had higher homopolymerization ability, and was thus more prone to forming a microblock structure. The statistical analysis of sequence-length distribution indicated that the number and length of cationic segments increased in the PAB molecules. In addition, the characteristic results of Fourier transform infrared (FTIR), proton nuclear magnetic resonance (^1^H NMR), and thermogravimetric analysis (TGA) provided evidence for the synthesis of copolymer with cationic microblocks. Finally, the results of dewatering tests demonstrated that sludge dewaterability was greatly improved by adding the synthesized novel flocculants, and the sludge-specific resistance to filtration, filter cake moisture content and residual turbidity all reached a minimum (68.7%, 5.4 × 1012 m·kg^−1^, and 2.6 NTU, respectively) at 40 mg·L^−1^. The PAB flocs were large, compact, difficult to break, and easy to regrow. Furthermore, PAB was more effective in the removal of protein from soluble extracellular polymeric substances (SEPSs). In summary, this study provides a novel solution to synthesize cationic microblock polyacrylamide for improving sludge dewatering.

## 1. Introduction

Nowadays, an ever-increasing amount of wastewater is treated using the biologically-activated sludge process, which results in a dramatic increase of waste sludge amount [1]. The management of the waste sludge is confronted with a crucial challenge due to high water content [2]. Therefore, sludge dewatering is regarded as an effective method before handling and disposal. Sludge is a complex colloidal system in which the dispersed particles at sizes around 10^−9^ m are always negatively charged due to the ionization of anionic functional groups [3]. The colloidal size and electrostatic repulsion interactions make the solid–liquid separation very difficult under natural conditions. Generally, chemical conditioning is often necessary prior to dewatering to aggregate small particles into bigger flocs.

A variety of chemical conditioning agents, such as Fenton’s reagents, surfactants, polyaluminum chloride and polyelectrolytes, are added to sludge in order to improve dewaterability [4]. Specifically, cationic polyacrylamides (CPAMs) have attracted extensive attention because of their higher efficiency, lower dosage, and lower impact on the environment [5,6,7]. The most concerning characteristics of CPAMs from the standpoint of dewatering efficiency are their molecular weight (MW) and charge density (CD). As the length of polymer molecular chain (i.e., the polymer molecular weight) increases, more particles are bounded by CPAM molecules, forming larger and stronger flocs that are easily separated by physical means. On the other hand, the CD has a large influence on charge neutralization effectiveness. The stability of the sludge colloidal system could be destroyed as a result of the reduced surface charge of the particles and hence a decreased electrical repulsion between them [8].

In recent years, the influence of the chemical structure, especially the block structure, of the polymer on dewatering performance has also been investigated. The polymer molecular chain with a random cation distribution often adopts a flatter adsorption on the surface of the particles, which reduces the opportunity for bridging interactions with other sludge particles and squanders the advantage of the long linear molecular chain. Inversely, the microblock structure limits the adsorption sites for the CPAM, which then adopts a more extended adsorbed configuration, resulting in effective bridging flocculation [9]. Template polymerization is a common method for the synthesis of CPAMs with block structure. Qingqing Guan et al. synthesized CPAMs through template polymerization and elucidated the effects of the template on the structural control of polymer [10]. Wei Chen et al. determined the optimal template polymerization parameters, such as molecular weight of template, the molar ratio of template to cationic monomer and systems pH and so on [11]. However, not only does this method require a huge number of templates, which influences the purity of the polymer, but the addition of template lowers molecular weight. To overcome the bottleneck, we introduced a new functional monomer to synthesize chemical conditioning flocculants with cationic microblock structures.

Surface-active monomers (surfmers) containing amphiphilic structure and polymerizable vinyl double bonds display unique physicochemical properties [6]. They can form organized molecular assemblies by self-assembly in aqueous solution, such as micelle, vesicle and lyotropic liquid crystal. In the process of surfmers and acrylamides copolymerization, the pre-assembly of surfmers will improve the ordered nature of the reaction system. Once the polymerization is initiated by an initiator, organized molecular assemblies will generate the block structure in polymers. Meanwhile, the most common hydrophilic groups are quaternary ammonium groups which can ionize to give cationic sites along the chain. Therefore, the process and reagents can be greatly simplified compared with template polymerization.

Free radical copolymerization in aqueous solution can be initiated by heat, rays and ultraviolet. The above-mentioned initiation processes have disadvantages, for instance, elevated temperature is used for breaking apart the chemical bonds and a radiation source which requires specific safety precautions is employed by photochemical initiation [12]. Ultrasound-initiated polymerization has a faster polymerization rate, narrower molecular weight distribution, higher monomer conversion and a smaller number of initiators than other initiation systems [13]. In addition, more stable uniform monomer assemblies get formed in ultrasound-initiated polymerization because of cavitational activity near the interface between aqueous phase and hydrophobic pocket by micro jets, which is conducive to the introduction of the cationic microblock structure. To the best of our knowledge, no report has attempted to study the synthesis and application of copolymers with cationic microblock structure in the sludge dewatering process involving the combined effect of surfmers and ultrasound.

In this study, the surfmer, benzyl dimethyl 2-(methacryloyloxy)ethyl ammonium chloride (BDMDAC), reported as forming micelles in aqueous solution, was adopted to prepare the P (AM-BDMDAC) (PAB) initiated by ultrasound with potassium persulfate as the initiator. Another monomer, methacrylatoethyl trimethyl ammonium chloride (DMC), was used to prepare the copolymer (P (AM-DMC) (PAD)) under the same synthetic conditions in that the DMC is similar to BDMDAC in structure but without self-assembly properties. In order to elucidate the effect of surfmer on cationic microblock structures, a comparison between PAB and PAD in this study was conducted. The reactivity ratios of monomers were calculated, and the sequence distribution of the molecules was statistically analyzed. The properties of polymers were characterized by Fourier transform infrared (FTIR), ^1^H NMR spectroscopy, and differential scanning calorimetry and thermogravimetric analysis (DSC–TGA). Finally, the sludge dewatering tests were carried out in terms of residual turbidity of the supernatant (RTS), filter cake moisture content (FCMC), specific resistance to filtration (SRF), flocs morphological properties and extracellular polymeric substance (EPS).

## 2. Materials and Methods

### 2.1. Materials

The acrylamide (AM), BDMDAC, and DMC used in this experiment were all of technical grade and were used without further purification. The monomer AM (98.5%, *w*/*w*) was obtained from Lanjie Tap Water Company (Chongqing, China); BDMDAC (80 wt % in water) was procured from Zibo Jinyin Chemical Co., Ltd. (Zibo, China); and DMC (80 wt % in water) was provided by Nanjing Jingrui Jiuan Biotechnology Co., Ltd. (Nanjing, China). Analytical grade urea (CO(NH_2_)_2_), hydrochloric acid (HCl), sodium hydroxide (NaOH) and potassium persulfate (K_2_S_2_O_8_) were purchased from Aladdin Co. Ltd. (Shanghai, China). All aqueous and standard solutions were prepared with deionized water supplied by a Milli-Q Plus water purification system (Millipore, Molsheim, France).

### 2.2. Preparation of Copolymer

Synthesis of the copolymer of AM and BDMDAC was carried out in a jacketed glass reactor and with a 13-mm stainless steel sonic wave emission probe. A 750-W (at 50% amplitude, 375 W) ultrasonic generator (Sonics Vibra-cell, Make: Ace) operating at a frequency of 22 kHz controlled by standard power source was used as an ultrasonic source to initiate the polymerization. The monomer solution was prepared by adding predetermined amount of AM (155.93 mmol) and BDMDAC (17.33 mmol) in distilled water to reach a monomer mass ratio of 40%, and it was then transferred to the reactor. Then, urea (2.0 wt % of total monomers mass) was added to increase the solubility and the pH of the reaction solution was adjusted to 4 (±0.2) by HCl and NaOH solution. Prior to the addition of the photoinitiator, the reaction vessel was purged with nitrogen gas (99.99%) for half an hour. Finally, the irradiation time under ultrasonics was set to 30 min (a shorter time than the photochemical initiation). After irradiation, a milk-white semitransparent solid was obtained which indicated the formation of copolymer. The products were purified with acetone and ethanol for several times and then dried in a vacuum oven at 60 °C for 2 days. PAD was prepared by the same synthesis process. The possible reaction mechanisms of PAB and PAD are shown in Supplementary Figure S1.

### 2.3. Determination of the Monomer Reactivity Ratio

The monomer reactivity ratio (*r*), namely, the ratio of chain radical homopolymerization rate constant to the copolymerization rate constant reflects the relative polymerization activity [14]. It was defined based on the following equations:

(1) $$r=\frac{k_{11}}{k_{12}}$$

where *k*_11_ and *k*_12_ are the rate constants of homopolymerization and copolymerization, respectively. When *r* is more than 1, the monomer tends to homopolymerize with the same monomers; otherwise, the monomer tends to copolymerize with other monomers. Besides, the larger the value is, the higher the relative activity will be. The monomer reactivity ratio is a crucial parameter to control polymer composition and structure in binary copolymerization [15].

In this experiment, two series of polymer products, each containing eight PAB or PAD samples, were prepared under the same conditions described in Section 2.2 except that the monomer conversions of all samples were controlled to less than 10%. Prior to synthesis, the reaction molar ratios of AM and cationic monomers were controlled at 9:1, 8:2, 7:3, 6:4, 5:5, 4:6, 3:7, 2:8 and 1:9. Afterward, the cationic degree of polymer was measured by colloid titration. The monomer reactivity ratios were calculated with Fineman–Ross, Kelen–Tüdö and Yezrielev−Brokhina−Roskin (Y−B−R) methods of which the detailed procedures are shown in Supplementary Text S1–S3 and Figures S1–S4 [16].

### 2.4. Composition and Sequence-Length Distributions of Polymers

The copolymer composition directly reflects the chemical structure of polymers; thus, the composition equation is used to evaluate instantaneous composition of the bipolymers in light of the following formula [17,18].

(2) $$F_{1}=\frac{r_{1}f_{1}^{2}+f_{1}f_{2}}{2a}$$

(3) $$F_{2}=1-\frac{\left(r_{1}-1\right){f_{2}}^{2}+\left(1-2r_{1}\right)f_{2}+r_{1}}{\left(r_{1}-2+r_{2}\right){f_{2}}^{2}+2\left(1-r_{1}\right)f_{2}+r_{1}}$$

where *F*_1_ and *F*_2_ are the molar ratios of AM and cationic monomer to the total copolymer units, respectively; *f*_1_ and *f*_2_ are the molar ratios of AM and cationic monomer to all material monomers before polymerization, respectively; and *r*_1_ and *r*_2_ are the monomer reactivity ratios obtained from Section 2.3.

Sequence-length distribution is referred to the monomer unit amount in its continuous chain segment and is important to characterize the block structure of the polymer [19,20,21]. In order to elucidate the influence of monomer self-assembly on the polymer microstructure, four feed ratios between AM and BDMDAC or DMC (8:2, 6:4, 4:6, 2:8) were adopted for statistical analysis. The details of the calculating equations are exhibited in Supplementary Texts S4 and S5.

### 2.5. Characterizations

The intrinsic viscosity of copolymer was measured by an Ubbelohde viscosity meter (Shanghai, China) equipped with a thermostatic water container (DKB-501S, Shanghai Jing Hong Laboratory Instrument Co., Ltd., Shanghai, China). Fourier transform infrared (FTIR) spectra of the dried copolymer samples were recorded with KBr pellets by a 550Series II infrared spectrometer (BRUKER Company, Zürich, Switzerland). The ^1^H NMR spectra were obtained in deuterium oxide (D_2_O) with an AVANCE 500 NMR spectrometer (BRUKER Company, Ettlingen, Germany). The thermal decomposition property of copolymer was determined by thermal gravimetric analysis (TGA) at a heating rate of 10 °C·min^−1^ under nitrogen flow of 20 mL·min^−1^ over a temperature range from 20 °C, up to 600 °C on a DTG-60H synchronal thermal analyzer (SHIMADZU, Kyoto, Japan).

### 2.6. Dewatering Tests

#### 2.6.1. Sludge Conditioning

The sludge samples were collected from secondary sedimentation tanks of the Jiguanshi Wastewater Treatment Plant in Chongqing, China. The initial sludge characteristics are listed in Table 1. A series of flocculants (shown in Table 2) were chosen to comparatively analyze the dewatering efficiency of new block copolymer. Experiments were carried out in 1500-mL beakers equipped with an electronic program-controlled jar test apparatus (TA6-4, Wuhan Hengling Technology Ltd., Wuhan, China) and the set dose of flocculants was added to each beaker containing 1000 mL of sludge. The mixture was then stirred at 200 rpm for 30 s, followed by 60 s of slow stirring at 40 rpm. Finally, flocs began to form and kept quiescent settling for 10 min.

#### 2.6.2. Determination of Sludge Dewaterability

The conditioned sludge was poured into Buchner funnel and filtrated under a constant pressure of 0.07 MPa. The volume of filtrate was measured by graduated flask which was used to calculate SRF. The resulting filter cakes were transferred to a crucible and dried at a thermostatic drying oven. FCMC and SRF were obtained according to the calculation method described in Supplementary Text S6 and S7, respectively. The residual turbidity was measured with HACH 2100N Turbidimeter (HACH Company, Loveland, CO, USA). The zeta potential of the sludge supernatant after dewatering was performed on the Zetasizer Nano ZS90 (Malvern Instruments Ltd., Malvern, UK).

#### 2.6.3. Characterization of Morphological Properties of Sludge

Dynamic sludge flocs size was monitored using a laser diffraction analyzer (Mastersizer2000, Malvern, UK) during the whole formation, breakage and re-growth process. d_50_ (50% of the flocs were of the sizes in the range 0–d_50_) was adopted to represent the average floc size. Floc strength and recovery factors were widely used to compare the degree of breakage and re-growth and were calculated as follows [22]:

(4) $$\mathrm{strength}\;\mathrm{factor}=\frac{B}{A}$$

(5) $$\mathrm{recovery}\;\mathrm{factor}=\frac{C-B}{A-B}$$

where *A*, *B* and *C* are the average flocs sizes in the steady phase before breakage, after the breakage period and after the re-growth to new steady phase, respectively.

The sludge cake after vacuum filtration was sampled and pretreated with spray gold after vacuum freeze drying. Then, the morphology of sludge was observed with a MIRA 3 LMU scanning electron microscopy (SEM) system (TES-CAN Company, Brno, Czech Republic).

#### 2.6.4. Extraction and Analysis of Soluble EPS (SEPS)

On the basis of previous work, the extraction and analysis method was designed described below [23]. A total of 80 mL of sludge suspension was taken using a tube (100 mL) and centrifuged at 3000× *g* for 15 min. The supernatant was carefully collected as SEPS of which the chemical oxygen demand (COD) was determined using HACH DR2800 spectrophotometer (HACH Company, Loveland, CO, USA) with 2 h of digestion. The polysaccharides and proteins from SEPS were measured by Anthrone method [24] and Lowry method [25], respectively.

## 3. Results and Discussion

### 3.1. Monomer Reactivity Ratios of the Polymers

**Figure 1:**
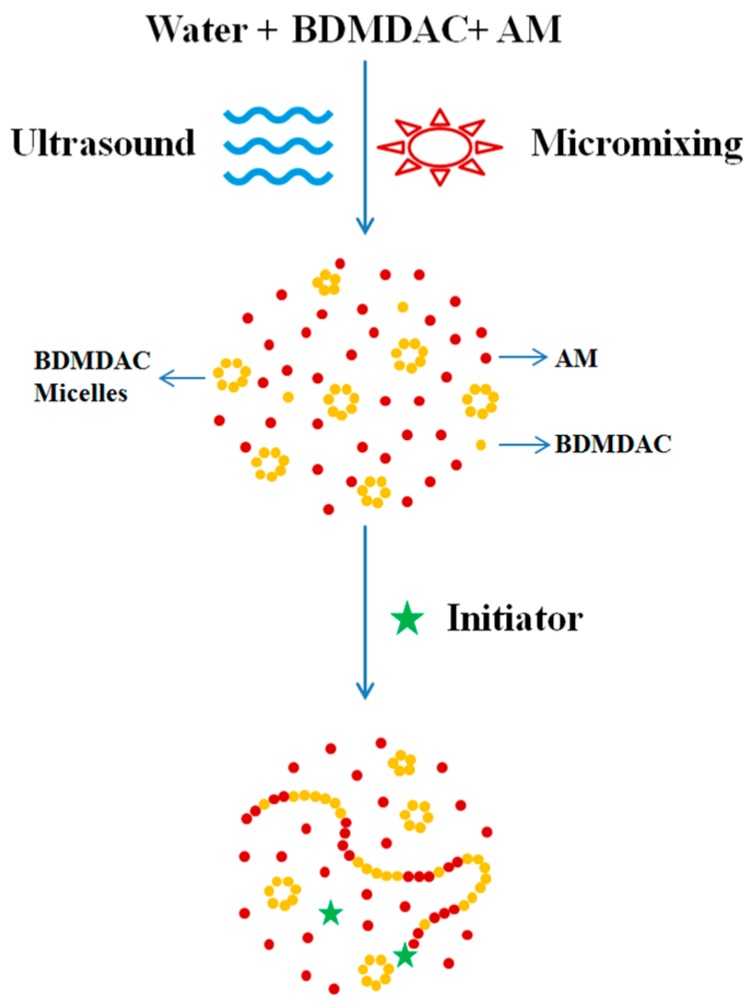
Schematic mechanism of the copolymerization.

The reactivity ratios of AM and cationic monomers calculated according to three methods are shown in Table 3 and the results were close to those of the previous study. In PAD copolymerization, *r*_AM_ and *r*_DMC_ were both lower than 1, which indicated the monomer copolymerization ability was stronger than that of homopolymerization [26]. Therefore, monomers inclined to copolymerize disorderly, forming shorter chain segments of same units in polymer molecule. As opposed to the above scenario, *r*_BDMDAC_ was greater than 1 in PAB system, which was attributed to stronger self-assembly ability of BDMDAC. Before polymerization reaction, BDMDAC could form micellar aggregation in the aqueous phase whereas DMC just assumed the dispersed state. The concentration of surfmers in micellar aggregation was similar to bulk polymerization, facilitating homopolymerization. When encountering a growing AM macroradical headgroup, they were embedded in polymer in the form of aggregation, of which the structure was regarded as a micro block. Accordingly, it was deduced that there were more and longer cationic blocks in the molecular chain of PAB than in PAD. Whereas, owing that AM reactivity ratio was less than 1, the PAB copolymer was composed of long BDMDAC segments embedded in short AM chain segments [27]. The above procedure is depicted in Figure 1.

### 3.2. Composition Equations of the Polymers

**Figure 2:**
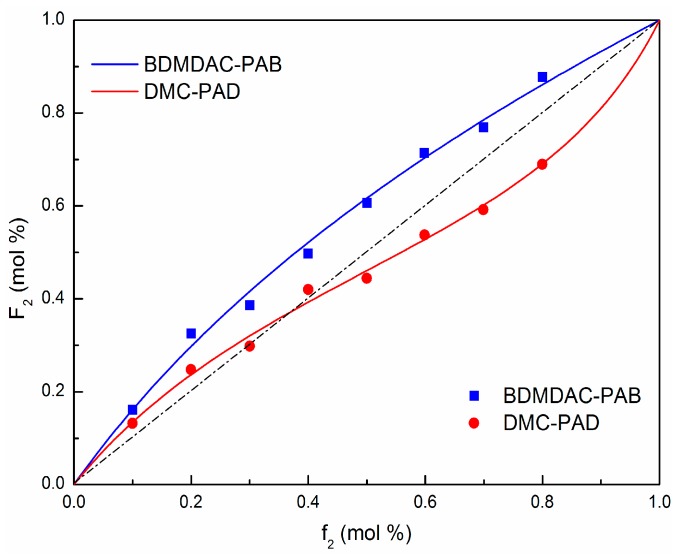
Composition curves and test results of the polymers. Note that the squares stand for the test results, the four solid curves are composition curves of the polymers, and the dashed line represents an ideal copolymerization.

The composition equations of the polymers were obtained on the basis of monomer reactivity ratios and feed ratios which are depicted in Figure 2 by nonlinear fitting, and the relationship of monomer composition between materials and copolymers was intuitively displayed. The curve of DMC-PAD accords with typical random copolymerization because the two monomers both tend to copolymerize, forming shorter homogeneous monomer chain segment. As shown in Figure 2, there was an azeotropic point at *f*_2_ (DMC−PAD) = 0.38, i.e., the feed molar ratio of DMC was higher than that in copolymers when beyond 0.38, otherwise it was the opposite. Moreover, we noticed that the curve of BDMDAC-PAB without intersecting the diagonal had no azeotropic point, which was in agreement with block copolymerization. The molar ratio of BDMDAC was invariably higher than the feed ratio, demonstrating a high utilization of cationic monomer. Furthermore, PAB would possess greater CD than PAD at same feed ratio since the PAB curve was always above PAD. Based on the above discussion, the new monomer not only enhanced the charge effect but also improved conversion.

### 3.3. Sequence Distributions of the Polymers

**Figure 3:**
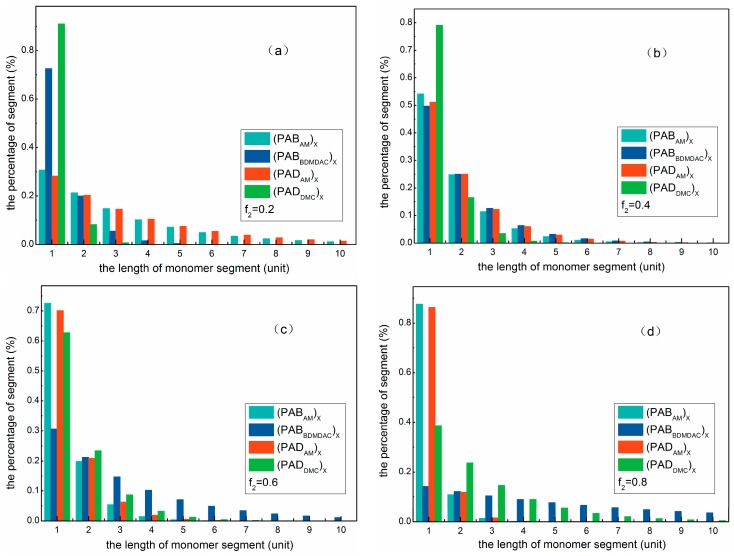
Sequence distributions of cationic monomer and acrylamide (AM) in the polymers under (**a**) *f*_2_ = 0.2; (**b**) *f*_2_ = 0.4; (**c**) *f*_2_ = 0.6; and (**d**) *f*_2_ = 0.8.

The sequence distributions of the monomer segments were in terms of microscopic perspective to evaluate molecular arrangements in comparison with macroscopical polymer composition equations. The results are summarized in Figure 3 and the detailed experimental data are listed in the Supplementary Tables S3–S6. As shown in Figure 3, the AM segments with the length of 1, 2, 3 in PAD (PAB) under *f*_2_ = 0.2 accounted for 28.22% (30.71%), 20.26% (21.28%), and 14.54% (14.74%), respectively. Correspondingly, the DMC (BDMDAC) segments with the length of 1, 2, 3 in PAD (PAB) accounted for 90.97% (72.49%), 8.21% (19.94%), and 0.7% (5.49%), respectively. This illustrated that the sequence distribution of cationic monomers was narrower with shorter chain segments, whereas AM sequence distribution was wider and longer under this condition. When the initial molar ratio of reactants was increased to 0.4 or 0.6, monomers in the two polymers tended to an alternate distribution along the molecular chain. Sequentially increased to 0.8, the situation of the AM and DMC (BDMDAC) segments was just the contrary to *f*_2_ = 0.2. Our results suggested that monomers sequence distributions were mainly dependent on *f*_2_, namely, feed ratio.

In addition, there were more long cationic chain segments in PAB than those in PAD under the same feed ratio. Besides, the length advantage became more and more obvious with the increase of feed ratio. This observation may be explained by the fact that BDMDAC monomers are inclined to form larger micelle aggregates at higher dosages, which contributed to longer cationic chain segments being embedded on encountering growing levels of macroradicals. The results are in agreement with the analysis of reactivity ratios and composition equations of polymers. In short, PAB had more and longer cationic blocks in the molecular chain than PAD.

### 3.4. Characterizations of the Polymers

#### 3.4.1. FTIR Spectra

**Figure 4:**
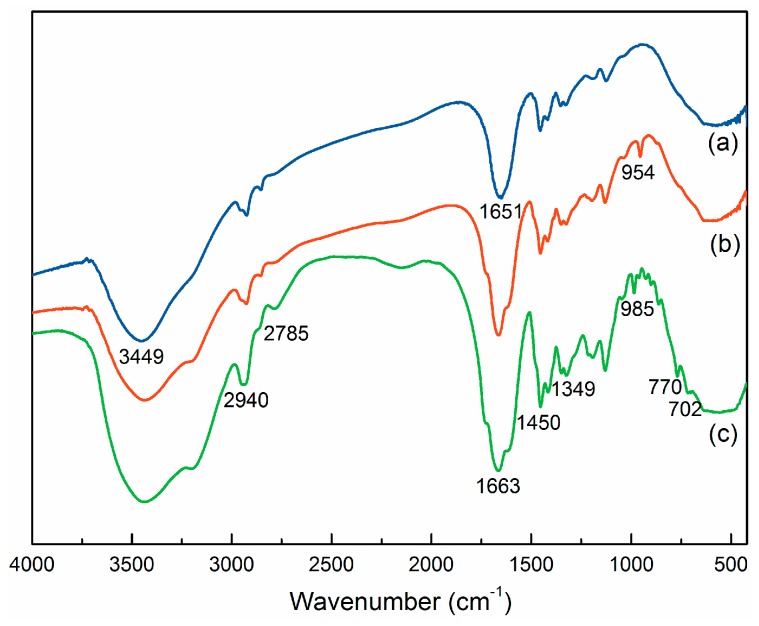
Fourier transform infrared (FTIR) spectra of (**a**) PAM; (**b**) PAD; (**c**) PAB.

The FTIR spectra of (a) PAM; (b) PAD and (c) PAB are shown in Figure 4. Strong absorption peaks were observed at 3449 and 1651 cm^−1^, which were ascribed to the strong stretching vibrations of the –NH and –C=O in the AM unit, respectively [28]. The characteristic absorption peaks at 1349 cm^−^^1^ were attributed to –CH_3_ bending vibrations of –N^+^(CH_3_)_2_^−^ in the BDMDAC unit, whereas the absorption peaks at 2940 and 2785 cm^−1^ were from the –CH_3_ and –CH_2_ asymmetric stretching vibrations, respectively [29]. Furthermore, the new adsorption peaks at 1450, 770 and 702 cm^−1^ of PAB were due to the benzene skeleton vibration in the BDMDAC unit [6]. The characteristic absorption peaks at 954 and 985 cm^−1^ were assigned to quaternary ammonium groups from DMC and BDMDAC, respectively. The spectral structures in Figure 2b,c were similar but had a slight shift in wavenumbers, indicating that the two polymers had similar chemical groups. In addition, the missing peaks at 770 and 702 cm^−1^ in PAD proved the lack of benzene ring, which was consistent with its theoretical chemical structure. Overall, the FTIR characterization results showed the formation of both copolymer PAB and PAD.

#### 3.4.2. ^1^H NMR Spectra

**Figure 5:**
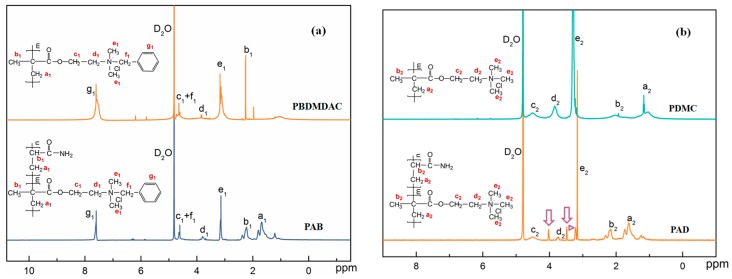
^1^H NMR spectra of the polymers: (**a**) poly(benzyl dimethyl 2-(methacryloyloxy)ethyl ammonium chloride) (PBDMDAC) and PAB; (**b**) poly(methacrylatoethyl trimethyl ammonium chloride) (PDMC) and PAD.

Figure 5 demonstrates the ^1^H NMR spectra of poly(benzyl dimethyl 2-(methacryloyloxy)ethyl ammonium chloride) (PBDMDAC), PAB, poly(methacrylatoethyl trimethyl ammonium chloride) (PDMC), and PAD, respectively, for convenient comparison and research. It could be seen from Figure 5a that the asymmetric peaks at δ = 1.69 ppm and δ = 2.23 ppm were associated with the protons of the backbone methylene (a_1_) and methine groups (b_1_), respectively. Protons in the two sequential methylene groups (c_1_, d_1_) connected to the ammonium group of BDMDAC were observed at δ = 4.64 and 3.79 ppm. The sharp peak at δ = 3.15 ppm was attributed to the protons of the two equivalent methyl (e_1_) at quaternary ammonium groups. The peaks at δ = 7.60 and 4.61 ppm were assigned to protons of the phenyl group (g_1_) and methylene (f_1_) connected to the phenyl group. The comparison between PBDMDAC and PAB revealed that most protons of cationic monomer appeared in PAB, which confirmed the successful copolymerization of BMDAC and AM. Moreover, the chemical shifts of adsorption peaks were very slight, indicating that the chemical environments of cationic monmer in PAB and homopolymer were almost identical. On the other hand, there are many split peaks marked with arrows in PAD compared with PDMC shown in Figure 5b. The result can be explained with the stereochemistry of the copolymers. PAD was a randomly distributed copolymer of AM and DMC, thus the adsorption peaks of cationic monomers were remarkably interfered with by adjacent protons generating more diverse chemical environments [30]. Nevertheless, the phenomena did not occur in PAB, which manifested that BMDAC was distributed in the form of block structure along the copolymer molecular chain.

#### 3.4.3. DSC−TGA

**Figure 6:**
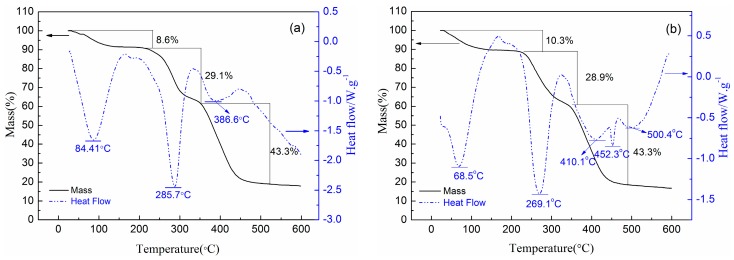
Differential scanning calorimetry and thermogravimetric analysis (DSC−TGA) curves of (**a**) PAD; and (**b**) PAB.

DSC−TGA analysis was often performed to estimate the thermal stability of products. In recent years, many researchers identified polymer microstructures using this method [31,32]. Figure 6 exhibited the DSC−TGA curves of PAD and PAB. Obviously, there were three thermal decomposition stages in both TGA curves, whereas the mass loss and temperature range were slightly different. The first stage occurred within the ranges of 30–185 °C with partial mass loss of 8.6% for PAD and 10.3% for PAB, respectively. These losses may result from water evaporation which was absorbed by powder samples with hydrophilic quaternary ammonium groups [33]. Accordingly, obvious endothermic peaks at 84.41 and 68.5 °C appeared on the DSC curves of PAD and PAB, respectively. The second stage ranged from 185–352 °C and 185–338 °C with partial weight loss 29.1% and 28.9% for PAD and PAB, respectively, which may be attributed to the thermal decomposition and imidization of the amide group and the abruption of methyl or benzyl in the two quaternary ammonium groups [34]. The two copolymers began to disintegrate in this stage and the initial temperature was 185 °C, manifesting the satisfactory thermal stabilities. The corresponding endothermic peaks were observed at 285.7 °C for PAD and 269.1 °C for PAB. During the third stage, weight losses of approximately 43.3% for PAD and PAB in the ranges of 352–522 and 338–490 °C, respectively, were observed. These could belong to thermal decomposition of the carbon backbone, leading to a sharp decline of the molecular weight and degree of polymerization. Specifically, there are three peaks at 410.1, 452.3, 500.4 °C in the DSC curves of PAB but just one in PAD. Many studies have reported that the phenomenon is related to the copolymer sequence distribution [35]. PAB, with BDMDAC blocks, AM blocks and a few random distributed monomers showing three different endothermic peaks due to the decomposition of these three types of backbones. However, the monomers were randomly distributed in the main chains of PAD and exhibited a combined degradation effect. These results of DSC−TGA thus further demonstrated the microblock structure of PAB.

### 3.5. Dewatering Test

#### 3.5.1. Effect of Sequence Distribution and Dosage on Dewatering Performance

**Figure 7:**
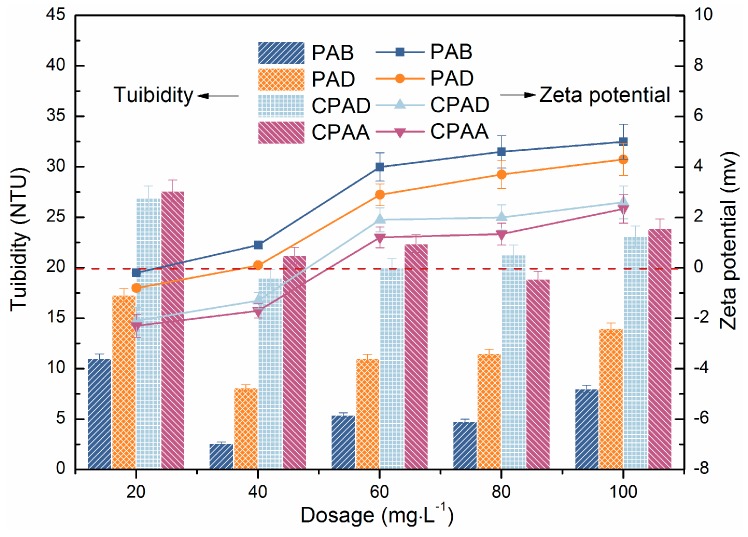
Effect of flocculants dosage on tuibidity and zeta potential of the sludge supernatant.

**Figure 8:**
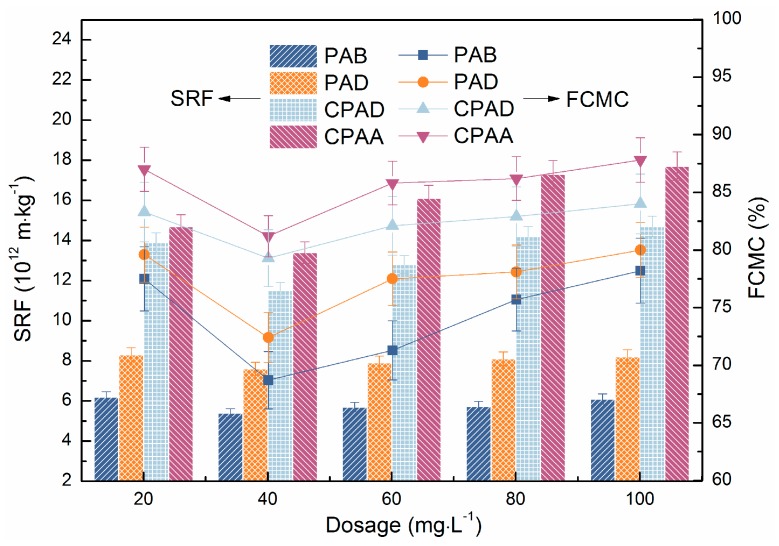
Effect of flocculants dosage on specific resistance to filtration (SRF) and filter cake moisture content (FCMC).

The above characterization results of two copolymers corroborate that there is a blocky distribution of cationic units in PAB and a random distribution in PAD. Therefore, to accurately evaluate the impact of cationic sequence distribution on sludge dewatering efficiency, four copolymers detailed in Table 2 were chosen for comparison. Figure 7 and Figure 8 presented the results of sludge conditioned by different flocculants. Four copolymers existed with a similar variation trend, which were in line with typical organic flocculant conditioning. It can be seen that sludge residual turbidity, FCMC and SRF decreased with increasing flocculant dosages and reached the lowest point at the optimal dosage, then increased when continuing increase dosages. From Figure 8 it can also be seen that sludge conditioned by PAB had the best dewatering performance; this indicates that the cationic microblock structure compared with random distribution is more propitious to sludge dewatering. Moreover, our prepared PAD preceded commercial products.

The zeta potential of the supernatant was a reasonable parameter in terms of interpreting flocculation mechanism [36]. As shown in Figure 7, the zeta potential of the conditioned sludge all climbed from the negative charge region to positive charge region with the increase of dosage, and that of PAB was always the highest at a given dosage. This finding indicated the charge neutralization mechanism played an important role in dewatering. Thus, the discrepancy of the dewatering performance might be attributed to various charge neutralization ability. The blocky distribution could concentrate cationic charges in the polymer molecular chain, which contributed to more sufficiently neutralizing the surface negative charge and compressing electrical double layer of sludge particles. Furthermore, the adsorption between cationic microblocks and particles was firmer. All these promoted the particles to aggregate to denser floccules, resulting in more moisture drainage. On the other hand, the zeta potential was not at the isoelectric point when reaching optimal dosage, which manifested in that the charge neutralization was not the only mechanism [37]. The block structure in PAB limited the adsorption sites for the sludge, which then adopted a more extended configuration, and the tails and loops stretching in the solution were long enough and in favour of capturing the sludge particles, leading to effective bridging flocculation and larger floccules. In contrast, the molecular chains of the copolymers (PAD, commercial poly (acrylamide -acryloxyethyltrimethyl ammonium chloride), commercial PAD), with a random cation distribution, could only adopt a flat adsorption on the particles surface, which reduced the opportunity for bridging interactions with other sludge particles [38]. Thus, the larger and denser sludge floccules conditioned by cationic microblocky flocculant (PAB) were more suitable for dewatering. In addition, better dewatering performance of PAD than commercial CPAM may be due to surface modification effect of ultrasound-initiated polymerization.

#### 3.5.2. Effect of Sequence Distribution on Morphological Properties of Sludge

##### Sludge Floc Size

**Figure 9:**
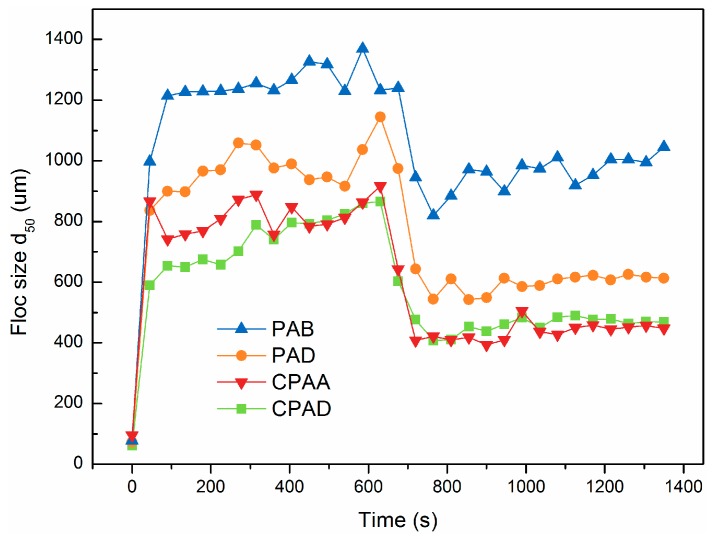
Effects of different sequence distribution on breakage and re-flocculation.

The dynamic sludge floc size during flocculation, breakage and re-flocculation stage of PAB, PAD, CPAD and CPAA at optimal dosage (40 mg·L^−1^) was depicted in Figure 9. After addition of four flocculants, original sludge floc size obviously increased and kept steady during slow agitation; when the external strong shear force was exerted, the sludge broke immediately then regenerated to a certain degree at subsequent slow stage. It should be noted that the size of sludge particles treated with PAB ranged from 1200 to 1400 um, which were the biggest among four flocculants. In previous research, floc size was known as an important index to determine dewaterability, and smaller flocs were always related to poorer dewaterability since they were more prone to plugging the filtration medium while bigger flocs can provide more voids for water release [39]. In addition, the strength factors (S_Fs_) of sludge flocs shown in Table 4, which reveal the ability of resistance to shearing, were consistent with the following order: PAB > PAD > CPAD > CPAA. This demonstrated that the sludge flocs generated by PAB could withstand stronger shear force and were less likely to break. In the re-flocculation stage, recovery factor (R_F_) of PAB was 34.15%, whereas the RFs of PAD, CPAD and CPAA were 12.36%, 13.84% and 7.59%, respectively, which demonstrated re-growth capability except when PAB was quite low.

As discussed in Section 3.5.1, the blocky distribution can enhance the bridging effect, whereas the randomly distributed structure cannot. In addition, previous studies suggested that floc strength was strongest by bridging flocculation [40]. Thus, more colloidal particles were tightly caught to form bigger and stronger flocs by cationic blocky copolymer (PAB). The low re-growth capability of PAD, CPAD and CPAA was attributed to the irreversible breakage of polymer molecular chains when exposed to high shear force. As for PAB, the blocky distribution could concentrate cationic charges in the polymer molecular chain; after the molecular chain breakage, the residual segmers adsorbed on particles surface had a fairly high charged density. These regions were called positive patches which can give negative particles electrostatic attraction and hence cause re-flocculation [9]. This observation coincided with the study results of Yoon and Deng et al. who found that re-flocculation after floc breakage occured more readily in the case of electrostatic patch than bridging [41].

##### Structural Characteristics of Sludge Cake

**Figure 10:**
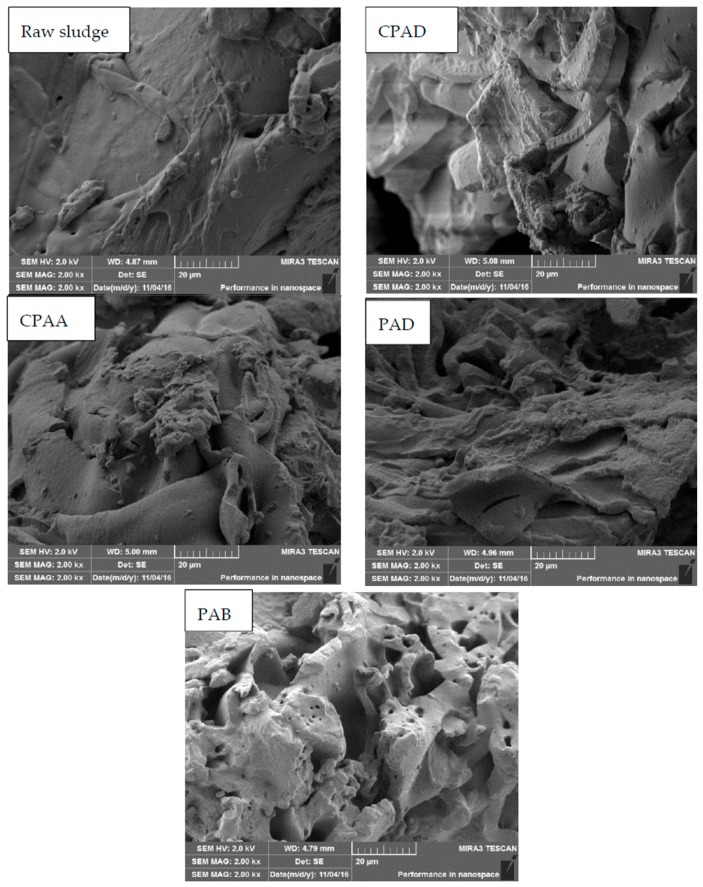
SEM images of sludge cake conditioned by different flocculants.

The filtration resistance of sludge dewatering was mainly from filter medium and cake. In recent years, much research has reported that sludge conditioned by polymers is highly compressible and more readily forms impermeable filter cakes, impeding further dewatering, which accounts for most of the total filtration resistance [4,42]. Thus, the structural characteristics of sludge cake were evaluated after vacuum filtration. The SEM images of sludge cake were demonstrated in Figure 10. Obviously, the raw sludge cake had a smooth structure with few channels and voids. It was responsible for compressible sludge particles deformed under vacuum pressure inducing cake void closure. It was noteworthy that there was a slight increase of pores after conditioning by PAD, CPAD and CPAA, while PAB had a complicated porous structure, providing more channels for drainage. The enhanced charge neutralization and bridging ability by the cationic blocky structure contributed to forming compact flocs, which can maintain original shape even under high pressure. In addition, the rigid structure of benzene ring in PAB may be also an important reason for reducing sludge cake compressibility.

#### 3.5.3. Effect of Sequence Distribution on Sludge Soluble EPS Properties

**Figure 11:**
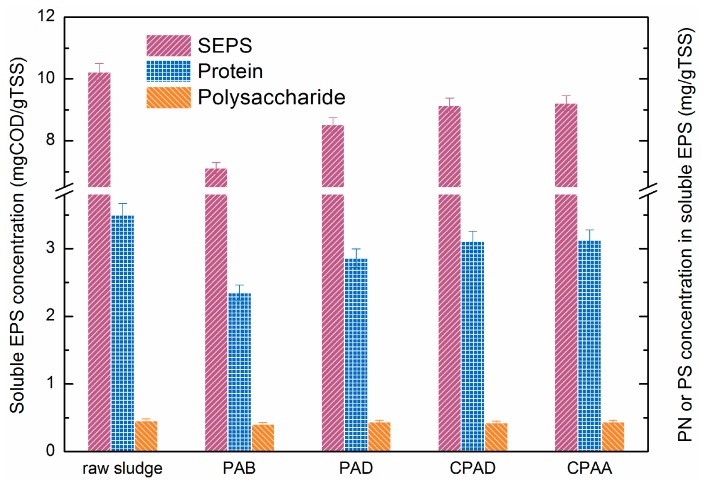
Effect of different flocculants on concentrations of SEPS and composition. SEPS: soluble extracellular polymeric substance

According to the previous reports, the sludge dewatering performance mainly depended on the soluble EPS (SEPS) chemical properties [43,44,45]. The variation of SEPS concentration and composition was assessed to unravel the mechanism of sludge conditioned by cationic microblock copolymer. As shown in Figure 11, the SEPS concentration of conditioned sludge compared to raw sludge was decreased. Low SEPS concentration was always related to the reduction of viscosity and low compressibility, consequently improving dewatering performance. Protein (PN) and polysaccharide (PS) were the dominant fractions of SEPS [46]. After conditioned by flocculants, the PN concentration declined, while the variation of PS was unconspicuous. Thus, the decrease of SEPS mainly derived from the removal of PN, which was flocculated to solidoid by CPAM. Many studies indicated that the ratio of PN and PS played a crucial role in estimating dewaterability and low protein/polysaccharide was suitable for the dewatering process [47,48]. Obviously, PDA with a cationic microblock structure was more effective in removal of PN at optimal dosage because of enhanced electrical neutralization and adsorption bridging ability. This result was aligned with the investigation of sludge morphological properties, namely, PDA had higher efficiency in sludge dewatering improvement.

## 4. Conclusions

In this study, a novel flocculant PAB with a cationic microblock structure was successfully synthesized by the ultrasound-initiated polymerization technique, which significantly improved the sludge dewatering performance. Based on the investigations, the main conclusions can be drawn as follows:

1. The reactivity ratio of monomers suggested that novel cationic monomer BDMDAC had higher homopolymerization ability, and thus are more prone to forming microblocks. The statistical analysis of sequence-length distribution indicated that the number and length of cationic segments were increased in the PAB molecules. In addition, the characteristic results of FTIR, ^1^H NMR, and TGA provided evidence for the synthesis of cationic microblock copolymer.
2. Sludge dewaterability was greatly improved by adding the synthesized novel flocculant and the sludge-specific resistance to filtration, filter cake moisture content and residual turbidity all reached a minimum (68.7%, 5.4 × 10^12^ m·kg^−1^, 2.6 NTU, respectively) at 40 mg·L^−1^. The excellent performance was associated with the combined effect of surfmers and ultrasound.
3. The bigger and more compact sludge flocs conditioned by PAB were not easy to break when exposed to high shear force. Furthermore, their re-growth capability was relatively high, benefiting from patch re-flocculation.
4. PAB with cationic microblock structure was more effective in removal of PN in SEPS because of enhanced electrical neutralization and adsorption bridging ability, which was conducive to reducing the sludge viscosity and compressibility.

## Figures and Tables

**Table 1:** Characteristics of the initial sludge without flocculant addition.

Indicator	pH	Moisture Content (%)	Zeta Potential (mv)	VSS/TSS (mg/L)	SRF (10^13^ m/kg)	Turbidity of Sludge Bulk (NTU)
Value	6.93 ± 0.12	98.2 ± 0.5	−18.9 ± 1.4	0.63 ± 0.02	5.6 ± 0.3	35.3 ± 1.7

VSS: volatile suspended solids; TSS: total suspended solids; SRF: specific resistance to filtration.

**Table 2:** Details of used flocculants in sludge conditioning test.

Flocculants	Intrinsic Viscosity (dL·g^−1^)	Average Molecular Weight (10^6^ Da)	Cationic Degree (%)
PAB	6.94	2.86	60
PAD	7.18	2.98	60
CPAA	7.22	3.00	60
CPAD	7.22	3.00	60

PAB: poly[acrylamide-benzyl dimethyl 2-(methacryloyloxy)ethyl ammonium chloride]; PAD: poly(acrylamide-methacrylatoethyl trimethyl ammonium chloride); CPAA: commercial poly (acrylamide-acryloxyethyltrimethyl ammonium chloride); CPAD: commercial PAD.

**Table 3:** Monomer reactivity ratios of the polymers.

Methods	PAD		PAB	
	*r*_AM_	*r*_DMC_	*r*_AM_	*r*_BDMDA*C*_
Fineman–Ross Method	0.64	0.42	0.55	1.47
Kelen–Tüdö Method	0.63	0.39	0.57	1.52
Y−B−R Method	0.64	0.39	0.57	1.56
Average	0.64	0.40	0.56	1.52

*r*: monomer reactivity ratio; DMC: methacrylatoethyl trimethyl ammonium chloride; BDMDAC: benzyl dimethyl 2-(methacryloyloxy)ethyl ammonium chloride; Y–B–R: Yezrielev−Brokhina−Roskin.

**Table 4:** Floc parameters of different flocculants under the same condition.

Flocculants	S_F_ (%)	R_F_ (%)
PAB	64.73	34.15
PAD	54.41	12.36
CPAA	49.76	7.59
CPAD	data	13.84

## Supplementary Materials

The following are available online at www.mdpi.com/1996-1944/10/3/282/s1. Scheme S1. Proposed reaction scheme of the synthesis of PAB and PAD, Text S1. Fineman–Ross method, Text S2. Kelen–Tüdö method, Text S3. Yezrielev–Brokhina–Roskin method, Text S4. The composition equations of PAB and PAD, Text S5. The calculating formulas of sequence distribution of PAB and PAD, Text S6. Analytical methods for filter cake moisture content (FCMC), Text S7. Analytical methods for sludge-specific resistance to filtration (SRF), Table S1. Fineman–Ross and Kelen–Tudos parameters for acrylamide (AM)/benzyl dimethyl 2-(methacryloyloxy)ethyl ammonium chloride (BDMDAC) copolymerization system, Table S2. Fineman–Ross and Kelen–Tudos parameters for AM/methacrylatoethyl trimethyl ammonium chloride (DMC) copolymerization system, Table S3. The sequence distributions of monomer segments of PAB and PAD under *f*_2_ = 0.2, Table~S4. The sequence distributions of monomer segments of PAB and PAD under *f*_2_ = 0.4, Table S5. The sequence distributions of monomer segments of PAB and PAD under *f*_2_ = 0.6, Table S6. The sequence distributions of monomer segments of PAB and PAD under *f*_2_ = 0.8, Figure S1. Determination of AM and DMC reactivity ratios by the Fineman–Ross method, Figure S2. Determination of AM and DMC reactivity ratios by the Kelen–Tüdö method, Figure S3. Determination of AM and BDMDAC reactivity ratios by the Fineman–Ross method, Figure S4. Determination of AM and BDMDAC reactivity ratios by the Kelen–Tüdö method
