# Supplementary material for: Improvement of Sludge Dewaterability by Ultrasound-Initiated Cationic Polyacrylamide with Microblock Structure: The Role of Surface-Active Monomers
Source: Materials (Basel). 2017 Mar 13;10(3):282. doi: 10.3390/ma10030282 (PMC5503336; doi:10.3390/ma10030282)
Supplement: Supplementary file 1 [file materials-10-00282-s001.pdf]

Cover Page for Supplementary

Manuscript title:

Improvement of Sludge Dewaterability by Ultrasound-Initiated Cationic Polyacrylamide with Microblock Structure: Role of Surface-Active Monomers

Author list:

Chuanliang Zhao [1,2], Huaili Zheng [1,2,*], Li Feng [1,2], Yili Wang [3], Yongzhi Liu [1,2], Bingzhi Liu [1,2] and Badradine Zakaria Djibrine [1,2]

[1] Key laboratory of the Three Gorges Reservoir Region's Eco-Environment, State Ministry of Education, Chongqing University, Chongqing 400045, P.R. China
[2] National Centre for International Research of Low-carbon and Green Buildings, Chongqing University, Chongqing 400045, P.R. China
[3] College of Environmental Science and Engineering, Research Center for Water Pollution Source Control and Eco-remediation, Beijing Forestry University, Beijing 100083, P.R. China
* Correspondence: zhl@cqu.edu.cn; Tel.: +86 23 65120827

Total number of pages (including the cover page):

8 pages

Contents:

Scheme S1. Proposed reaction scheme of the synthesis of poly[acrylamide-benzyl dimethyl 2-(methacryloyloxy)ethyl ammonium chloride] (PAB) and poly(acrylamide-methacrylatoethyl trimethyl ammonium chloride) (PAD)

Text S1. Fineman–Ross method

Text S2. Kelen–Tüdö method

Text S3. Yezrielev–Brokhina–Roskin method

Text S4. The composition equations of PAB and PAD

Text S5. The calculating formulas of sequence distribution of PAB and PAD

Text S6. Analytical methods for filter cake moisture content (FCMC)

Text S7. Analytical methods for sludge-specific resistance to filtration (SRF)

Table S1. Fineman–Ross and Kelen–Tudos parameters for acrylamide (AM)/ benzyl dimethyl 2-(methacryloyloxy)ethyl ammonium chloride (BDMDAC) copolymerization system

Table S2. Fineman–Ross and Kelen–Tudos parameters for AM/methacrylatoethyl trimethyl ammonium chloride (DMC) copolymerization system

Table S3. The sequence distributions of monomer segments of PAB and PAD under f_2 = 0.2

Table S4. The sequence distributions of monomer segments of PAB and PAD under f_2 = 0.4

Table S5. The sequence distributions of monomer segments of PAB and PAD under f_2 = 0.6

Table S6. The sequence distributions of monomer segments of PAB and PAD under f_2 = 0.8

Figure S1. Determination of AM and DMC reactivity ratios by the Fineman–Ross method

Figure S2. Determination of AM and DMC reactivity ratios by the Kelen–Tüdö method

Figure S3. Determination of AM and BDMDAC reactivity ratios by the Fineman–Ross method.

Figure S4. Determination of AM and BDMDAC reactivity ratios by the Kelen–Tüdö method.

Scheme:

Scheme S1. Proposed reaction scheme of the synthesis of PAB and PAD.

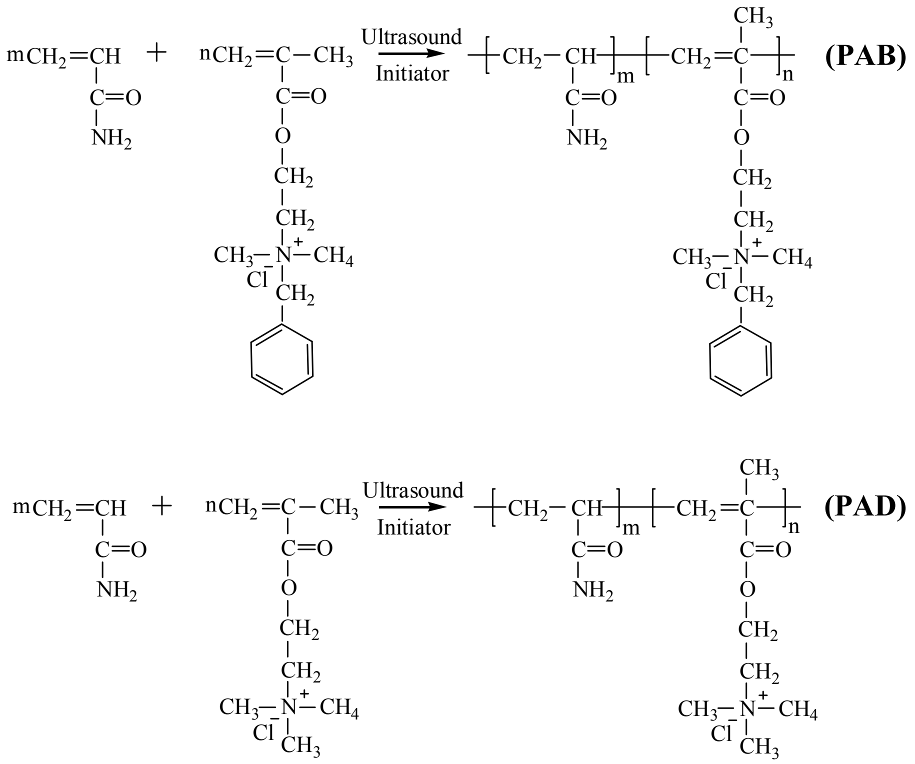

Text:

Text S1. Fineman–Ross method.

$$\mathbf{G = r_{AM}H - r_{CM}}$$

$$\mathbf{G = \frac{R(\rho - 1)}{\rho};\ H = \frac{R^2}{\rho}} \qquad \mathbf{(S1)}$$

In the above formulae, r_{AM} is the reactivity ratio of AM monomer in AM and cationic monomer pair, r_{CM} is analogous to r_{AM}, R is the molar ratio of AM and cationic monomers in the raw material before the polymerization reaction, and ρ is the molar ratio of AM and cationic monomers in the polymers at low conversion. After the G and H of each point were obtained, the linear fitting curve relating to G and H could be plotted, and the r_{AM} and r_{CM} can be obtained through the slope and intercept of straight line.

Text S2. Kelen–Tüdö method.

$$\mathbf{\eta = (r_{AM} + \frac{r_{CM}}{\delta})\xi - \frac{r_{CM}}{\delta}}$$

$$\mathbf{\eta = \frac{G}{\delta + H};\ \xi = \frac{H}{\delta + H};\ \delta = \sqrt{H_{min} \times H_{max}}} \qquad \mathbf{(S2)}$$

η and ξ could be obtained based on the results of Fineman–Ross method and formula S2. Then, the linear fitting curve relating to η and ξ could be plotted, and the r_{AM} and r_{CM} were calculated through the slope and intercept of straight line.

Text S3. Y–B–R method.

$$\mathrm{r_{AM}} = \frac{\mathrm{A_2C_1 + nC_2}}{\mathrm{A_1A_2 - n^2}}, \qquad \mathrm{r_{CM}} = \frac{\mathrm{A_1C_2 + nC_1}}{\mathrm{A_1A_2 - n^2}}$$

$$\mathrm{A_1} = \sum_{\mathrm{i=1}}^{\mathrm{n}} \frac{X_\mathrm{i}^2}{\mathrm{Y_i}};\ \mathrm{A_2} = \sum_{\mathrm{i=1}}^{\mathrm{n}} \frac{\mathrm{Y_i}}{\mathrm{X_i^2}};\ \ \mathrm{C_1} = \sum_{\mathrm{i=1}}^{\mathrm{n}} \mathrm{X_i}(1 - \frac{1}{\mathrm{Y_i}});\ \mathrm{C_2} = \sum_{\mathrm{i=1}}^{\mathrm{n}} \frac{\mathrm{Y_i}}{\mathrm{X_i}}(\frac{1}{\mathrm{Y_i}} - 1) \qquad \text{(S3)}$$

In the above formulae, n is the number of test groups; X is the molar ratio of AM to cationic monomers before polymerization; and Y is the molar ratio of AM units to cationic units in copolymers at low conversion.

Text S4. The composition equations of PAB and PAD.

$$\boldsymbol{F_{BDMDAC-PAB} = 1 - \frac{-0.44f_{BDMDAC}^2 - 0.12f_{BDMDAC} + 0.56}{0.08f_{BDMDAC}^2 + 0.88f_{BDMDAC} + 0.56}} \qquad \textbf{(S4)}$$

$$\boldsymbol{F_{DMC-PAD} = 1 - \frac{-0.36f_{DMC}^2 - 0.28f_{DMC} + 0.64}{-0.96f_{DMD}^2 + 0.72f_{DMC} + 0.64}} \qquad \textbf{(S5)}$$

In the above formulae, F is the molar ratio of the units of one monomer to the total copolymer units, and f is the molar ratio of one monomer to all material monomers before polymerization.

Text S5. The calculating formulae of sequence distribution of PAB and PAD.

$$\boldsymbol{(p_1)_x = p_{11}^{x-1}(1 - p_{11}),} \qquad \boldsymbol{(p_2)_x = p_{22}^{x-1}(1 - p_{22})}$$

$$p_{11} = \frac{r_{AM}(1 - CD)}{CD}, \qquad p_{22} = \frac{r_{CM}CD}{1 - CD} \qquad \text{(S6)}$$

In the above formulae, x is the number of monomer units in the segment. $(\mathrm{p_1})_\mathrm{x}$is the probability of generating the xAM segment which possesses x successive AM monomer units, it equals to the percentage of xAM segments in all AM segments in the copolymer. $(\mathrm{p_2})_\mathrm{x}$ is analogous to $(\mathrm{p_1})_\mathrm{x}$; and $\mathrm{p_{11}}$ is the probability of generating the 2AM segment which possesses two successive AM monomer units. $\mathrm{p_{22}}$ is analogous to $\mathrm{p_{11}}$.

Text S6. Analytical methods for FCMC.

The filter cake was placed into a crucible and dried for 24 h at 105 °C in a thermostatic drying oven. FCMC can be calculated by formulae S7:

$$\mathrm{FCMC} = \frac{M_1 - M_2}{M_1 - M_0} \qquad \text{(S7)}$$

where M_1 is the total weight of the filter cake without drying and crucible, M_2 is the total weight of the filter cake after drying and crucible, and M_0 is the weight of the crucible.

Text S7. Analytical methods for SRF.

The SRF of sludge can be calculated using formulae S8.

$$\mathrm{SRF} = \frac{2bPA^2}{\mu C} \tag{S8}$$

where P is the filtering pressure (N/m²), A is the filtering area (m²), μ is the kinetic viscosity (N s/m²), b is the slope of the filtration curve (S9), and C is the filter cake weight per unit volume filter (kg/m³), which can be obtained by formulae 10.

$$\frac{t}{v} = bv + a \tag{S9}$$

where t is the filtering time (s), and v is the filtrate volume (m³).

$$\mathrm{C} = \frac{\frac{1}{C_i}}{100 - C_i} - \frac{C_f}{100 - C_f} \tag{S10}$$

where C_i is the moisture content of the initial sludge, and C_f is the moisture content of the filter cake.

Table:

Table S1. Fineman–Ross and Kelen–Tudos parameters for AM/BDMDAC copolymerization system initiated by ultrasound.

No.	R	ϱ	G	H	ζ	η	δ
1	9	5.22	7.28	15.52	0.85	0.40	2.67
2	4	2.07	2.07	7.73	0.75	0.20	
3	2.33	1.59	0.86	3.41	0.56	0.14	
4	1.5	1.01	0.01	2.23	0.46	0.003	
5	1	0.65	-0.54	1.54	0.37	-0.13	
6	0.67	0.4	-1.01	1.12	0.30	-0.27	
7	0.43	0.3	-1.00	0.62	0.19	-0.31	
8	0.25	0.14	-1.54	0.45	0.15	-0.50	

Table S2. Fineman–Ross and Kelen–Tudos parameters for AM/DMC copolymerization system initiated by ultrasound.

No.	R	ϱ	G	H	ζ	η	δ
1	9	6.57	7.63	12.33	0.90	0.56	1.31
2	4	3.04	2.68	5.26	0.80	0.41	
3	2.33	2.35	1.34	2.31	0.64	0.37	
4	1.5	1.38	0.41	1.63	0.55	0.14	
5	1	1.25	0.20	0.80	0.38	0.09	
6	0.67	0.86	-0.11	0.52	0.29	-0.06	
7	0.43	0.69	-0.19	0.27	0.17	-0.12	
8	0.25	0.45	-0.31	0.14	0.10	-0.21	

Table S3. The sequence distributions of monomer segments of PAB and PAD (1: AM, 2: cationic monomer) under f_2 =0.2.

x	$(P_{PAB1})_x$	$(P_{PAB2})_x$	$(P_{PAD1})_x$	$(P_{PAD2})_x$
1	0.31	0.72	0.28	0.91

2	0.21	0.20	0.20	0.08
3	0.15	0.05	0.15	0.01
4	0.10	0.02	0.10	0
5	0.07	0.01	0.07	0
6	0.05	0	0.05	0
7	0.03	0	0.04	0
8	0.02	0	0.03	0
9	0.02	0	0.02	0
10	0.01	0	0.01	0

Table S4. The sequence distributions of monomer segments of PAB and PAD (1: AM, 2: cationic monomer） under f_2 =0.4.

x	$(P_{PAB1})_x$	$(P_{PAB2})_x$	$(P_{PAD1})_x$	$(P_{PAD2})_x$
1	0.54	0.50	0.51	0.79
2	0.25	0.25	0.25	0.17
3	0.11	0.13	0.12	0.03
4	0.05	0.06	0.06	0.01
5	0.02	0.03	0.03	0
6	0.01	0.02	0.01	0
7	0.01	0.01	0.01	0
8	0	0	0	0
9	0	0	0	0
10	0	0	0	0

Table S5. The sequence distributions of monomer segments of PAB and PAD (1: AM, 2: cationic monomer) under f_2 =0.6.

x	$(P_{PAB1})_x$	$(P_{PAB2})_x$	$(P_{PAD1})_x$	$(P_{PAD2})_x$
1	0.73	0.31	0.70	0.63
2	0.20	0.21	0.21	0.23
3	0.05	0.15	0.06	0.09
4	0.02	0.10	0.02	0.03
5	0	0.07	0.01	0.01
6	0	0.05	0	0.01
7	0	0.03	0	0
8	0	0.02	0	0
9	0	0.02	0	0
10	0	0.01	0	0

Table S6. The sequence distributions of monomer segments of PAB and PAD (1: AM, 2: cationic monomer） under f_2 =0.8.

x	$(P_{PAB1})_x$	$(P_{PAB2})_x$	$(P_{PAD1})_x$	$(P_{PAD2})_x$
1	0.88	0.14	0.86	0.39
2	0.11	0.12	0.12	0.24
3	0.01	0.10	0.02	0.15
4	0	0.09	0	0.09
5	0	0.08	0	0.05

6	0	0.07	0	0.03
7	0	0.06	0	0.02
8	0	0.05	0	0.01
9	0	0.04	0	0.01
10	0	0.04	0	0

Figure:

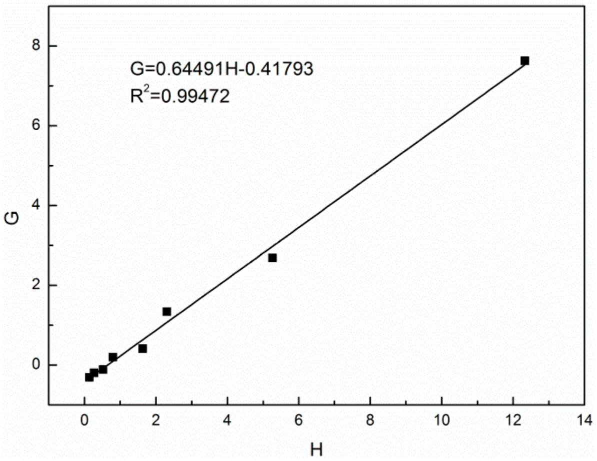

Figure S1. Determination of AM and DMC reactivity ratios by the Fineman–Ross method.

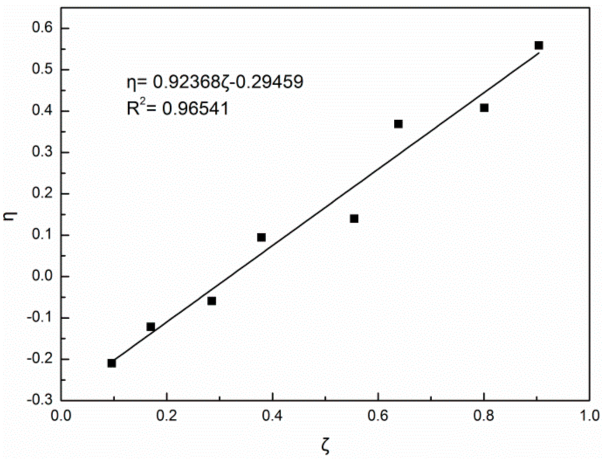

Figure S2. Determination of AM and DMC reactivity ratios by the Kelen–Tüdö method

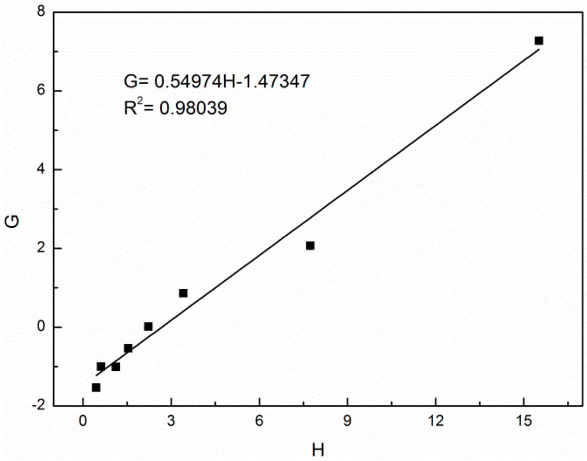

Figure S3. Determination of AM and BDMDAC reactivity ratios by the Fineman–Ross method.

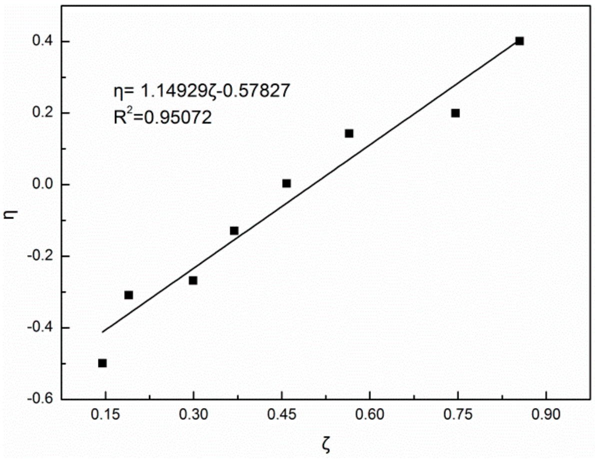

Figure S4. Determination of AM and BDMDAC reactivity ratios by the Kelen–Tüdö method.
